# 4040 Investigation of a Series of 1,4-diaryl-pyrazolo-pyridinones as Anti-Leishmanial Agents

**DOI:** 10.1017/cts.2020.75

**Published:** 2020-07-29

**Authors:** Hannah Noel Corman, Douglas A. Shoue, Bruce J. Melancon, Mary Ann McDowell

**Affiliations:** 1 University of Notre Dame; 2 Eck Institute of Global Health, University of Notre Dame; 3 Warren Center Drug Discovery & Development, University of Notre Dame

## Abstract

OBJECTIVES/GOALS: This study was conducted in order to identify novel chemical compounds that exhibit anti-leishmanial activity and to further characterize their efficacy and toxicity in *in vitro* and *in vivo* systems in the hopes of future chemotherapeutic developments. METHODS/STUDY POPULATION: We developed a novel, target-free fluorometric high-throughput screen (HTS) to identify small molecules with anti-leishmanial activity. Screening of 10,000 small molecules from the ChemBridge DIVERset-EXP library cassette #5 yielded 210 compounds that killed 80% of parasites. One hundred nine (109) molecular scaffolds were represented within the hit compounds, including the 1,4-diaryl-pyrazolo-pyridinone (1,4-DAPP). A total of 27 novel 1,4-DAPP compounds were synthesized and anti-leishmanial efficacy and host cell toxicity was determined using L. donovani mCherry expressing amastigotes and THP-1 macrophages. Additional pharmacokinetic analyses of a potent 1,4-DAPP compound were conducted. RESULTS/ANTICIPATED RESULTS: Four experimental compounds had IC50 values less than 5μM, providing similar anti-leishmanial activity to miltefosine. Compound 9279817 had a clearance almost twice the rate of normal hepatic blood flow and had a relatively high volume of distribution, indicating this compound is rapidly cleared and distributes into tissues. *in vitro* rat liver microsome assays suggest a rapid metabolism of 9279817, and MS/MS results suggest this metabolite is most likely formed via oxidation of the sulfur on the lower aromatic ring. DISCUSSION/SIGNIFICANCE OF IMPACT: This study revealed a novel structural class of compounds that have anti-leishmanial activity. *in vitro* experiments show compounds with similar efficacy as miltefosine while having significantly less toxicity, suggesting that this class could be further developed as a potential chemotherapeutic.

